# Psychometric characteristics of the Spanish version of the HIV Symptom Index

**DOI:** 10.1186/s41687-024-00780-2

**Published:** 2024-10-01

**Authors:** Olatz Ibarra-Barrueta, Oihana Mora-Atorrasagasti, Itziar Palacios-Zabalza, Urko Aguirre-Larracoechea, Maria Jose Legarreta, Nerea González-Hernández

**Affiliations:** 1 Department of Pharmacy, Hospital de Galdakao-Usansolo, Barrio Labeaga 46A, Galdakao, 48960 Spain; 2 Research Unit, Hospital de Galdakao-Usansolo, Galdakao, Spain

**Keywords:** HIV infections, Anti-HIV agents, Questionnaire, Adverse effects, Psychometrics, Patient reported outcome, PROM

## Abstract

**Background:**

The aim of this study was to determine the psychometric properties of the Spanish-language version of the HIV-Symptom Index (HIV-SI) questionnaire in Spanish patients undergoing antiretroviral therapy.

**Methods:**

Between 2014 and 2016, an observational, multicenter, prospective cohort study was conducted in seventeen Spanish hospitals to validate HIV-SI questionnaire in terms of: construct validity (confirmatory factor analysis), internal consistency (Cronbach’s alpha), convergent validity (Pearson’s correlation coefficient) and Known-group validity. In addition, a sensitivity to change analysis was also performed.

**Results:**

A total of 232 patients were included in the study. They had a mean age of 46.17 (SD9.82) and were 75% male. The median overall score for the HIV-SI was 10 (IQR 4– 19.5) and the most common symptoms reported were feelings of nervousness or anxiety, fatigue or energy loss, feeling sad or depressed, stomach pain or bloating, and difficulty sleeping. In the current study, the Spanish HIV-SI questionnaire showed a high internal consistency (α = 0.89) and adequate construct validity (CFI and TLI > 0.90). When contrasted with the MOS-HIV questionnaire, an inverse correlation was found. It showed a good association with the mental (*r*=-0.61; *P* < 0.0001) and physical score (*r*=-0.60; *P* < 0.0001). In a multivariate analysis, the age of the patient, female condition, hepatitis C coinfection, concomitant treatment and non-adherence resulted in a higher HIV-SI score.

**Conclusions:**

Our study has shown that the Spanish HIV-SI is a valid and reliable self-administered PROM for routine measurement of patient- reported symptoms among Spanish patients on antiretroviral treatment.

**Supplementary Information:**

The online version contains supplementary material available at 10.1186/s41687-024-00780-2.

## Background

High-activity antiretroviral therapy (ART) has made an important contribution to controlling Human Immunodeficiency Virus (HIV) infection by significantly reducing its morbimortality [1]. However, it requires a high level of adherence to the prescribed treatment. The existence of adverse side-effects has proved to be a determining factor, and one that is clearly associated with lack of treatment adherence, principally because such effects are strongly associated with the patient’s quality of life [2–6]. In the AdiCONA study, patients with suboptimal adherence reported significantly higher levels of treatment-related symptoms and side effects than adherent patients [4].

Patient-reported outcome (PRO) is reported directly by the patient without interpretation of the patient’s response by a clinician or anyone else [7]. Several studies have shown that reporting of adverse effects differs depending on whether they are assessed by the patient or his/her physician (8–9), and according to the assessment method used. Structured questionnaires are considered to provide more information than open-ended questions [10]. Physicians tend to under-estimate mild and moderate adverse effects, focusing more on serious events. Moreover, a study by Modayil [9] found a 39.7% prevalence of adverse effects when subjects were closely monitored, as compared to 10.8% in the case of spontaneous reporting, demonstrating the importance of proactive follow-up of adverse effects.

Few patient-reported outcome measures (PROMs) have been developed to assess symptoms reported by patients on ART. The Memorial Symptom Assessment Scale (MSAS) and its short version MSAS-SF, [11] measures the prevalence, frequency, severity, and discomfort associated with physical and psychological symptoms over the previous week [12–13]. This questionnaire is validated in patients with cancer, however it has not been properly validated among People Living With HIV/AIDS (PLWHA) population. A Visual Analogue Scale (VAS) is one of the scales used for rating pain and is often employed in epidemiologic and clinical research to measurthe intensity or frequency of several symptoms. In PLWHA, VAS has been used to measure current health status and adherence, [14] however, it has not been used to measure symptoms properly.

There are only two questionnaires validated in PLWHA. Holzemer’s questionnaire, “Revised Sign and Symptom Checklist for HIV (SSC-HIVrev)” [15], consists of 72 questions and takes into account patient-reported symptoms and their intensity in the previous 24 h [16]. Although the SSC-HIVrev is validated in PLWHA, it is very extensive and only reflects symptoms reported in the past 24 h.

Justice’s “HIV Symptom Index” (HIV-SI) [17], has been validated among PLWHA on ART, and its use is supported by the American Adult AIDS Clinical Trials Unit Outcomes Committee (AACTG), which also refers to it as the “Symptoms Distress Module” (SDM) [18]. Several studies have used it, either in its entirety or with subsequent modifications to match the specific purposes of each study [18–22].

Justice’s HIV-SI questionnaire consists of 20 questions on specific HIV symptoms obtained from a review of the literature and the opinion of experts. The questionnaire considers symptoms reported by patients and their intensity, measured on a scale of 4 options. It measures symptoms that have occurred within the past four weeks. It is easy to apply and presents a consistent and significant correlation with the physical and mental dimensions of the Medical Outcomes Survey HIV (MOS-HIV) scale, and with severity of the disease [17]. Finally, although it has been translated into Spanish by the AACTG, it has not been properly validated in the Spanish population.

The main objective of this study was to determine the psychometric characteristics of the Spanish version of the HIV-SI questionnaire among PLWHA on treatment and its sensitivity to change. In addition, we tried to establish the relationship between adherence, sociodemographic and clinical variables and symptoms reported by patients.

## Methods

An observational, multicenter, prospective cohort study was conducted to validate the HIV-SI questionnaire in the Spanish PLWHA population on antiretroviral treatment, in an outpatient clinic setting, using MOS-HIV as a gold standard.

After a clinician’s review and cognitive interview amongst a group of 17 patients to assess the comprehensiveness and comprehensibility of the HIV-SI questionnaire items, we added a further question to the twenty in the original test, to include any other symptom that the patient considered appropriate. First, a pilot phase was completed in 75 patients in 5 hospitals to confirm a consistent and significant correlation of HIV-SI with the MOS-HIV questionnaire [24]. Then, the study was extended to another 12 hospitals.

The inclusion criteria for pilot and main study were as follows, adults with HIV infection aged over 18 on antiretroviral treatment for at least one month, who gave written consent, came in person to pick up the medication from the hospital pharmacy department and without cognitive impairment.

Patients were recruited prospectively and consecutively in the pharmacy department of 17 hospitals in Spain. The study phase began in 2014 and consisted of 2 visits, with an interval of between 6 months and one year between them. The data collection phase of the study ended in 2016 and patient information was obtained from the patient’s medical record but also by asking the patient directly at the study visit.

The following questionnaires were used for the study: HIV-SI, MOS-HIV, Simplified Medication Adherence Questionnaire (SMAQ) and VAS. All paper- based questionnaires were self-administered at the same time, in the first and second visit.

In 10% of the patients, after 7 days from the first visit, the HIV-SI questionnaire was re-administered again to assess test-retest reliability, with a transitional question to evaluate whether there has been any change in the patient’s condition. At a second visit, the patient completed all PROMs again to assess the sensitivity to change of the HIV-SI test, together with a qualitative question to detect any other changes that might affect the results.

The HIV-SI questionnaire seeks to compile symptoms occurring in the previous four weeks and symptom distress. Each question or symptom was rated on a five- point scale from 0 to 4 (0 = do not have symptoms; 1 = have symptoms, but no bother; 2 = have symptoms, little bother; 3 = have symptoms, bother; 4 = have symptoms, bothers me a lot). The HIV-SI score was calculated by totaling the scores for each item. The final score ranges from 0 to 80, as a unidimensional scale, with higher values indicating a greater symptom distress. As in the original validation study, for dichotomized analyses, a bothersome symptom was defined as a symptom that was reported as “bothers me” or “bothers me a lot” [17].

Additionally, two VAS scales were included to measure patients’ experience with symptoms or adverse effects. The VAS Tolerance Scale (VTS) measured the level of tolerance (where 0 meant very bothersome and 10 meant no discomfort at all). The VAS Frequency Scale (VFS) assessed the number of days with poor tolerance, from none to all.

The MOS-HIV questionnaire was used as a gold standard to determine the convergent validity of the Spanish version of HIV-SI. MOS-HI is an HIV-specific measure of health-related quality of life (HRQOL) validated in the Spanish HIV population [25] and has been widely used as a gold standard in the validation of many questionnaires. It has good psychometric properties with high internal consistency and cross-cultural validity [26]. The MOS-HIV questionnaire has 35 items grouped into the following ten dimensions: mental health; quality of life; health distress; cognitive function; energy/fatigue; overall health; role function; physical function; pain and social function. It also provides a physical and mental health subscale, scored from 0 to 100 with higher scores reflecting better perceived health.

Antiretroviral treatment adherence was calculated using the SMAQ questionnaire [27]. This questionnaire has six questions for measuring adherence. Nonadherence is defined as being an affirmative answer to any of the qualitative questions, more than two doses missed over the past week, or over 2 days of total non-medication during the past 3 months.

Pharmacy refill record in the previous three or six months was also assessed to calculate the medication possession ratio (MPR), and non-adherence was defined as an MPR of below 95% [28, 29].

Sociodemographic and clinical variables were collected from the medical record, including variables related to the treatment. The CD4 cell counts and HIV viral load (VL) closest to the day of the survey was selected. Viral suppression or undetectable VL was considered if below 50 copies/mL.

The study protocol was approved by the Institutional Review Board of the Basque Country and by the local committee at each hospital. The Spanish Agency for Medicines and Health Products (AEMPS), considered as a post-authorization study of observational prospective follow-up. Patients’ written consent was required for recruitment.

### Statistical analysis

At least 100 patients were required to validate the 20-item questionnaire (5 per item). Assuming a 20% non-response, the final required sample size was found to be 125. If any question remained unanswered, the entire questionnaire was invalidated, and the patient was excluded from the validation study.

The analysis of the results was performed descriptively as a whole and stratified according to patient adherence. Means, medians, standard deviations and interquartile ranges were calculated for quantitative variables and frequencies and percentages for qualitative variables. For the comparison of quantitative variables, the student’s t-test/ANOVA or the corresponding nonparametric tests were used if the continuous variables do not follow a normal distribution. For the association between categorical variables, the Chi-square test or Fisher’s exact test were used if the expected frequencies are less than 5.

To evaluates the construct validity of the HIV-SI questionnaire, confirmatory factor analysis (CFA) was applied. The objective was to assess the extent to which our data matched the one-factor model derived from the initial study [17]. The assessment of goodness-of-fit was conducted using the subsequent indices: (i) The Chi-square (χ^2^) test was performed by dividing the number of degrees of freedom (DF); a result below 2 was deemed acceptable [30]; (ii) The root mean squared error of approximation (RMSEA); a value below 0.10 indicated a satisfactory fit [31]; (iii) The Tucker-Lewis Index (TLI) and the Comparative fit index (CFI); values exceeding 0.90 were deemed acceptable fits. The internal consistency was assessed by means of the Cronbach’s alpha coefficient and the McDonald’s omega coefficient, where a value of > 0.70 was considered acceptable. For the test-retest reliability the intraclass correlation coefficient (ICC) was used. A high ICC score indicated that the questionnaire had a high test-retest reliability.

Convergent and divergent validity were determined by means of Pearson’s bivariate correlation analysis with bilateral contrast, comparing the questionnaire to be validated with MOS-HIV questionnaires as a gold standard. Differences by groups of patients according to the variables collected in the study, such as CD4 level, presence of hepatitis and type of treatment were measured, to assess the known-group validity.

Sensitivity to change was evaluated by comparing HIV-SI difference from baseline and using Cohen effect size [32] in those groups of patients who change or switch treatment.

Finally, a generalized multivariate linear model was developed to determine the predictive factors that could have influence in the total symptoms score. As explanatory variables, those that in the univariate analysis had obtained a p-value less than 0.20 were considered [33]. All these statistical procedures were performed with the SAS System V9.2 statistical package, assuming statistical significance when *p* < 0.05.

## Results

### Descriptive analysis

**Fig. 1:**
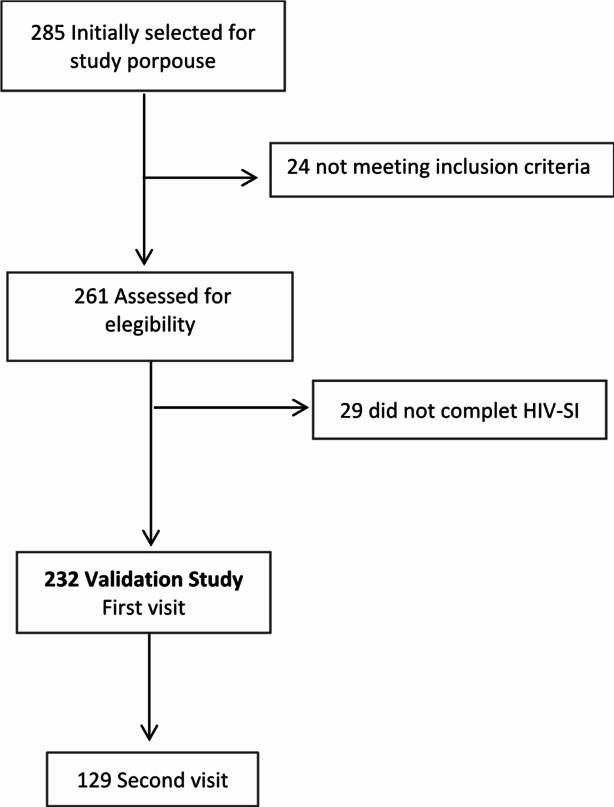
Patients flow chart

A total of 285 patients in 17 hospitals in Spain were initially selected for participation, twenty-four patients were excluded because they did not meet the inclusion criteria and 29 patients did not complete any item in the questionnaire (Fig. 1). Only 232 patients were finally included in the first visit and 129 in the second visit. Pretreated patients were found to be more likely to not have completed the HIV-SI questionnaire compared to naive patients (Supplementary Table 1).

The sociodemographic and clinical characteristics of the study sample are shown in Table 1. The mean age in our study cohort was 46.17 years (± SD 9.82), mostly male and treatment-experienced patients with undetectable viral load and CD4 count of over 200 cells/ mm^3^. Most patients (80%) were on treatment with triple combination therapy, mainly with a combination of two nucleoside reverse transcriptase inhibitors (NRTI) and another drug. More than 50% had other concomitant treatments in addition to antiretroviral therapy and 3% were on treatment for the hepatitis C virus (HCV).

**Table 1:** Patients demographic and clinical characteristics at baseline (*N* = 232)

		*n* (%)	Mean (± SD)/ Median [IQR25-75]
**Sociodemographic variables**			
Age (years)	<43	77 (33.19)	46.17 (± 9.82)
	>=43 y < = 50	77 (33.19)	
	> 50	78 (33.62)	
Sex assigned at bird	Male	174 (75)	
	Female	58 (25)	
Education level	No studies	12 (5.17)	
	Elementary school	83 (35.78)	
	High school	59 (25.43)	
	University degree or more	59 (25.43)	
	Unknown	19 (8.19)	
Work situation	Working	119 (51.29)	
	No working	105 (45.26)	
	Unknown	8 (3.45)	
Transmission route	MSM	67 (28.88)	
	Heterosexual	66 (28.45)	
	IDU	56 (24.14)	
	Other: mother- child, transfusion	5 (2.15)	
	Unkown	38 (16.38)	
**Clinical variables**			
Duration of infection years (*n* = 212)			12.37 (± 8.77)
CD4 cell count, cells/mm^3^	< 200	19 (8.19)	663.52 (± 348.48)
	200–499	64 (27.59)	
	>= 500	149 (64.22)	
HIV VL	Undetectable	208 (89.66)	
	Detectable	24 (10.34)	
CDC	A	109 (46.98)	
	B	21 (9.05)	
	C	41 (14.67)	
	Unkown	61 (26.29)	
HBV coinfection	No	166 (71.55)	
	Yes	60 (25.86)	
	Unkown	6 (2.59)	
**Treatment variables**			
Treatment experience	Naive	62 (26.72)	
	Experienced	165 (71.12)	
	Unkown	5 (2.15)	
Years taking antiretroviral therapy			6,87 [3.14–14.57]
Years taking current ART treatment (*n* = 202)			3.17 (± 2.78) 2.56 [0.83–4.91]
Type of ART therapy	2NRTI + NNRTI	66 (28.4)	
	3NRTI	56 (24.1)	
	2NRTI + PI	50 (21.6)	
	2NRTI + INSTI	14 (6)	
	bPI monotherapy	12 (5.2)	
	Dual therapy	15 (6.5)	
	Others	19 (8.2)	
Number of ART daily pills			2.77(± 1.66)
ART daily intake	Bid	52 (22.41)	
	Qd	180 (77.59)	
Others concomitant treatment HCV treatment		120 (51.72) 7 (3.02)	
SMAQ- patient adherent		131(56.47)	
MPR- Adherence level MPR	MPR ≥ 90%	197 (84.91)	95.19 (± 8.09)
	MPR ≥ 95%	171 (73.71)	
	MPR ≥ 100%	122 (52.59)	
	Median measuring interval in days		180 [180–294]

*ART* antiretroviral therapy; *MSM* men who have sex with men; *IDU* intravenous drug user; *VL* viral load; *bid* twice a day; *qd* once a day; *IQR* interquartile range; *SD* standard deviation; *MPR* medication possession ratio; *SMAQ* Simplified Medication Adherence Questionnaire; *NRTI* nucleoside reverse transcriptase inhibitors; *NNRTI* non-nucleoside reverse transcriptase inhibitor; *bPI* boosted protease inhibitor; *INSTI* integrase strand transfer inhibitor

The study group showed a mean MPR of 95.19% (SD 8.09). On the other hand, the SMAQ questionnaire classified 56.47% patients as adherent.

The median overall score of the HIV-SI questionnaire was 10 (IQR 4–19.5). The median number of symptoms reported for patients was 5.5 (IQR 3–9), but the median bothersome symptoms was 0 (IQR 0–3).

The symptoms most often reported by patients were feeling nervous or anxious (110; 47.41%); fatigue or energy loss (105; 45.26%); feeling sad, down, or depressed (105; 45.26%); bloating, pain, or gas in stomach (102; 43.97%); difficulty sleeping (101; 43.53%). Among bothersome symptoms, the most common were nervousness and anxiety (45; 19.40%), followed by difficulty sleeping (41; 17.67%); muscle aches or joint pain and stomach pain (both with 35 responders; 15.09%); sadness (33; 14.22%); and problems having sex (29; 12.50%). Only 27 (11.64%) patients reported having no symptoms at all, and 115 (49.57%) reported having no bothersome symptoms.

### Reliability or internal consistency

The results of the reliability analysis showed a Cronbach’s α of 0.89 and a value of 0.88 in McDonald’s omega for the initial 20 items in the HVI-SI draft, above the minimum value of 0.70. This datum was sufficient to confirm the internal consistency of the test.

Test-retest data were used to assess the stability of the HIV–SI at two different points of time. The ICC coefficient was 0.88, showing that HIV-SI scores were stable across time.

### Confirmatory factor analysis

The confirmatory analysis confirmed the original structure of the questionnaire. Results regarding the CFA indicated adequate goodness-of-fit of the structure of the questionnaire. The ratio between the χ2 Value (288.037) and the DF (170) was found to be < 2, leading to confirming its original structure. Moreover, CFI and TLI values were 0.921 and 0.911 respectively, both above 0.9, indicating satisfactory fit indexes. The RMSEA was 0.055 (0.044–0.065), below 0.10, indicating an adequate fit. Finally, standardized factor loadings ranged from 0.44 to 0.74 (Table 2).

**Table 2:** Confirmatory factor analysis for HIV-SI: item descriptive statistics, standardized factor loadings, parameter estimates and internal consistency

Symptom Item	Mean ± SD	Standardized estimates of factor loadings		Estimates of factor loadings (SE)	Cronbach’s Alpha	McDonald’s Omega
Main factor					0.89	0.88
HIV-SI1	0.91 ± 1.17	0.74	1.00 (0.0)			
HIV-SI2	0.37 ± 0.87	0.68	0.93 (0.10)			
HIV-SI3	0.41 ± 0.83	0.66	0.90 (0.09)			
HIV-SI4	0.55 ± 1.02	0.61	0.83 (0.08)			
HIV-SI5	0.75 ± 1.15	0.55	0.74 (0.09)			
HIV-SI6	0.36 ± 0.82	0.57	0.77 (0.08)			
HIV-SI7	0.58 ± 1.04	0.52	0.71 (0.10)			
HIV-SI8	0.96 ± 1.25	0.63	0.86 (0.07)			
HIV-SI9	1.11 ± 1.34	0.68	0.93 (0.08)			
HIV-SI10	1.0 3 ± 1.34	0.60	0.82 (0.09)			
HIV-SI11	0.69 ± 1.10	0.50	0.68 (0.09)			
HIV-SI12	0.44 ± 0.90	0.69	0.94 (0.08)			
HIV-SI13	0.57 ± 1.10	0.64	0.87 (0.09)			
HIV-SI14	0.33 ± 0.80	0.61	0.82 (0.10)			
HIV-SI15	0.98 ± 1.25	0.62	0.85 (0.09)			
HIV-SI16	0.86 ± 1.26	0.73	0.99 (0.08)			
HIV-SI17	0.76 ± 1.22	0.49	0.67 (0.10)			
HIV-SI18	0.70 ± 1.18	0.48	0.65 (0.10)			
HIV-SI19	0.32 ± 0.81	0.66	0.90 (0.09)			
HIV-SI20	0.39 ± 0.88	0.44	0.60 (0.15)			
Chi-square (DF)	288.037 (170)					
CFI/TLI	0.921/ 0.911					
RMSEA (90% CI)	0.055 (0.044–0.065)					

*DF* degrees of freedom; *CFI* Comparative fit index; *RMSEA* The root mean squared error of approximation; *SD* standard deviation; *SE* standard error; *TLI* Tucker-Lewis Index

### Convergent validity

The HIV- SI questionnaire was contrasted with MOS- HIV questionnaire. We found an inverse correlation between the HIV-SI and MOS-HIV questionnaires, with a statistically significant Pearson correlation coefficient in the overall score but also in the physical and mental health subscale score of the MOS-HIV, as shown in Table 3. In all dimensions, including pain, fatigue, physical, cognitive and mental health, the correlation was statistically significant (*p* < 0.0001), except for health transition (*p* = 0.837).

**Table 3:** Questionnarie’s convergent and divergent validity (*N* = 232)

	Mean (± SD)	Median (IQR P25-P75)	Pearson Correlation
**HIV- SI (0–80)**			
Total score	13.07 (± 11.73)	10 (4-19.50)	-
**MOS-HIV (0- 100)**			
General health	58.60 (± 24.69)	60 (40–75)	-0.51 (< 0.0001)
Physical function	87.86 (± 17.52)	100 (83.33–100)	-0.57 (< 0.0001)
Role function	90.00 (± 26.60)	100 (100–100)	-0.23 (0.0005)
Cognitive function	80.65 (± 22.37)	85 (70–100)	-0.53 (< 0.0001)
Pain	83.16 (± 22.84)	100 (66.67–100)	-0.64 (< 0.0001)
Mental health	72.02 (± 19.26)	76 (60–88)	-0.51 (< 0.0001)
Energy / fatigue	68.98 (± 20.76)	70 (55–85)	-0.54 (< 0.0001)
Health distress	81.79 (± 21.27)	90 (70–100)	-0.48 (< 0.0001)
Social function	91.75 (± 16.75)	100 (80–100)	-0.41 (< 0.0001)
Quality of life	66.14 (± 20.85)	75 (50–75)	-0.35 (< 0.0001)
Health transition	58.55 (± 18.65)	50 (50–75)	-0.01 (0.837)
Physical health	55.023 (± 7.86)	58.01 (51.18–60.62)	-0.61 (< 0.0001)
Mental health	51.32 (± 9.32)	53.40 (46.61–57.97)	-0.60 (< 0.0001)
**VAS (0–10)**			
VTS	8.68 (± 1.82)	9 (8–10)	-0.36 (< 0.0001)
VFS	1.31 (± 2.48)	0 (0–2)	0.36 (< 0.0001)

*HIV-SI* HIV Symptoms Index; *MOS-HIV* Medical Outcomes Survey HIV; *VAS* Visual Analogue Scale; *VTS* VAS Tolerance Scale; *VFS* VAS Frequency Scale; *SD* standard deviation; *IQR* interquartile range

Table 4 shows the results of the MOS-HIV test for each symptom. There is a significant difference in the MOS-HIV score between patients who reported symptoms and those who did not, also in bothersome symptoms. This difference is found in both the mental and the physical health dimension.

**Table 4:** Association between symptom absent or present in HIV–SI and MOS-HIV physical and mental health score

	MOS-HIV physical health score						MOS-HIV mental health score					
	Symptom			Symptom bothersome			Symptoms			Symptoms bothersom		
	Absent	Present	*p*-valor	Absent	Present	*p*-valor	Absent	Present	*p*-valor	Absent	Present	*p*-valor
1. Fatigue or loss of energy	57.87(5.31)	50.24(9.58)	< 0.0001	56.26(6.19)	42.91(11.41)	< 0.0001	54.93(7.19)	46.14(9.38)	< 0.0001	52.99(7.80)	38.29(8.27)	< 0.0001
2. Fever, chills, sweats	55.73(7.39)	48.78(10.31)	< 0.0001	55.28(7.41)	39.51(12.03)	< 0.0001	52.20(8.82)	45.71(9.71)	< 0.0001	51.68(8.80)	38.96(10.84)	0.0001
3. Feeling dizzy or lightheade	56.18(6.68)	49.30(10.50)	< 0.0001	55.07(7.54)	40.96(15.52)	0.0027	52.67(8.70)	45.62(9.21)	< 0.0001	51.48(9.00)	38.64(9.98)	0.0016
4. Pain, numbness or tingling in the hands or feet	56.32(6.58)	49.83(10.54)	< 0.0001	55.60(6.89)	42.84(13.07)	< 0.0001	52.25(8.64)	47.98(10.26)	0.0014	51.95(8.65)	41.57(10.80)	< 0.0001
5. Trouble remembering	56.55(6.75)	50.73(9.74)	< 0.0001	55.44(7.30)	46.07(12.15)	< 0.0001	53.70(8.30)	46.17(9.10)	< 0.0001	51.89(8.72)	43.43(10.86)	0.0002
6. Nausea or vomiting	55.85(6.82)	49.46(11.34)	0.0006	55.24(7.31)	37.68(14.46)	0.0004	52.11(8.49)	46.78(11.34)	0.0069	51.61(8.81)	36.80(12.37)	0.0017
7. Diarrhea or loose bowel movements	55.83(7.43)	51.54(9.48)	0.0005	54.94(8.08)	50.60(9.77)	0.0119	52.36(9.10)	47.96(9.03)	0.0003	51.48(9.24)	46.64(8.76)	0.0177
8. Felt sad, down or depressed	56.68(6.70)	51.76(9.48)	< 0.0001	56.22(6.55)	46.10(11.01)	< 0.0001	55.16(7.48)	46.05(8.95)	< 0.0001	53.49(7.49)	39.35(8.36)	< 0.0001
9. Felt nervous or anxious	57.37(5.80)	51.57(9.47)	< 0.0001	56.46(6.42)	47.72(10.60)	< 0.0001	55.08(6.95)	46.81(9.52)	< 0.0001	53.56(7.67)	42.15(9.09)	< 0.0001
10. Difficulty falling or staying asleep	56.69(6.77)	51.58(9.41)	< 0.0001	56.24(6.60)	46.76(10.85)	< 0.0001	53.98(7.50)	47.14(9.97)	< 0.0001	52.95(7.92)	42.49(10.09)	< 0.0001
11. Skin problems, such as rash, dryness or itching	56.10(6.87)	51.48(10.05)	< 0.0001	55.26(7.48)	47.30(12.36)	0.0003	52.65(8.21)	47.95(10.44)	0.0008	51.67(8.53)	44.83(13.32)	0.0172
12. Cough or trouble catching your breath	56.19(6.63)	49.12(10.82)	< 0.0001	55.44(7.04)	38.45(12.37)	< 0.0001	52.55(8.17)	46.20(10.94)	< 0.0001	51.75(8.60)	38.58(11.81)	< 0.0001
13. Headache	56.10(6.91)	49.19(10.76)	< 0.0001	55.48(7.27)	42.95(12.56)	< 0.0001	52.53(8.80)	46.15(9.49)	< 0.0001	51.89(8.82)	41.16(10.08)	< 0.0001
14. Loss of appetite or change in the taste of food	55.72(6.82)	47.96(12.39)	0.0004	54.85(8.01)	44.10(13.09)	0.0171	52.04(8.56)	45.59(11.24)	0.0009	51.26(9.17)	44.18(11.37)	0.0608
15. Bloating, pain or gas in your stomach	57.02(5.80)	51.26(10.02)	< 0.0001	56.22(6.28)	44.79(11.81)	< 0.0001	52.80(7.94)	48.71(10.44)	0.0030	52.36(8.22)	43.38(11.47)	< 0.0001
16. Muscle aches or joint pain	58.15(4.48)	48.65(9.84)	< 0.0001	56.40(6.32)	44.97(10.94)	< 0.0001	54.00(7.45)	46.60(10.06)	< 0.0001	52.63(8.15)	43.72(10.90)	< 0.0001
17. Problems with having sex, such as loss of interest or lack of satisfaction	55.82(6.74)	51.69(10.58)	0.0058	55.51(7.42)	47.69(11.06)	< 0.0001	53.06(8.01)	46.82(10.37)	< 0.0001	52.23(8.58)	43.06(10.06)	< 0.0001
18. Changes in the way your body looks such as fat deposits or weight gain	56.06(6.93)	51.68(10.01)	0.0004	55.55(7.55)	48.40(10.37)	< 0.0001	52.50(8.60)	48.38(10.06)	0.0017	52.09(8.91)	44.91(9.47)	< 0.0001
19. Problems with weight loss or wasting	55.98(6.85)	47.74(11.14)	< 0.0001	55.15(7.46)	43.68(14.33)	0.0006	52.46(8.46)	44.62(10.36)	< 0.0001	51.58(8.92)	42.03(11.21)	0.0009
20. Hair loss or changes in the way your hair looks	55.02(7.96)	51.79(9.90)	0.0143	54.58(8.47)	51.99(7.12)	0.0669	51.55(9.18)	48.36(9.55)	0.0272	51.19(9.25)	47.27(9.98)	0.0816
**Total**	59.17(3.98)	53.73(8.71)	0.0003	58.53(4.30)	50.56(9.53)	< 0.0001	56.89(6.30)	50.07(9.43)	0.0003	55.16(6.15)	46.88(10.04)	< 0.0001

Results as mean (SD)

According to the VAS scales, in VTS the median tolerance level was 9 (IQR 8–10) and the median number of days with poor tolerance was 0 (IQR 0–2) in VFS. There was also a moderate correlation between VAS scales and HIV-SI questionnaire (*p* < 0.0001).

### Known- groups validity (criterion related validity)

In a univariate analysis, age, being female, being unemployed or retired, transmission route, HCV coinfection, treatment-experience, concomitant treatment, and non-adherence to therapy reported significantly worse tolerance or more symptoms (Table 5). Years from diagnosis and years on antiretroviral treatment also negatively influenced tolerance.

**Table 5:** HIV-SI score across sociodemographic or clinical variables

Variables		HIV-SI score *N* = 232 Mean(SD); median[IQR_25 − 75_]	*P* valor
Age (years)	< 43	9.221(7.35); 7[3–15]	0.008
	>=43 y < = 50	16.77(14.46); 12[5–26]	
	> 50	13.24(11.15); 11[4–19]	
Sex assigned at birth	Male	11.91(10.54); 9[3–18]	0.025
	Female	16.57(14.28); 12[7–27]	
Work situation	No working	15.61(13.89); 11[4–27]	0.016
	Working	10.36(8.65); 9[3-116]	
Transmission route	IDU Drugs	11.40(9.89); 9[3–17]	0.029
	Others	16.86(14.10); 13.50[6-26.50]	
HCV coinfection	No	11.19(10.29); 9[3–16]	0.001
	Yes	17.92 (13.77); 14.50[8–26]	
Treatment	Naive	9.60(8.63); 7[2–16]	0.006
	Experienced	14.70(12.45); 11[5–22]	
Others concomitant treatment	No	10.13(9.59); 8[3–16]	0.001
	Yes	15.58(12.99); 12[5–25]	
SMAQ adherence	No	16.20(13); 13[5–25]	< 0.001
	Yes	10.66(10.05); 8[2–16]	
Duration of infection (years)	<=5	8.41(7.96); 6[3–12]	0.001
	5–10	13.23(9.13); 13[7-17.5]	
	10–20	16.64(13.30); 5[6–25]	
	> 20	15.58(13.86); 10[5.5–25]	
Years taking antiretroviral therapy	<=5	9.60(8.85); 7[3–16]	0.002
	5–10	13.40(11.92); 12[5–18]	
	10–20	17.89(13.65); 15.5[8–28]	
	> 20	13.47(11.64); 9[5–20]	

*IDU* intravenous drug user; *IQR* interquartile range; *SD* standard deviation; *SMAQ* Simplified Medication Adherence Questionnaire

Non-adherent patients by SMAQ had a significantly higher median score in the HIV-SI test than adherent patients [13 (IQR 5–25) vs. 8 (IQR 2–16), *p* < 0.001]. In contrast, MPR was not statistically associated with symptoms reported (*p* = 0.329).

Clinical variables related to disease severity, such as CD4 cell count, and viral load did not show a significant difference in HIV-SI score. This is probably related to the small size of the sample group of severe patients. Even so, there was a tendency towards more symptom distress reported among patients with a detectable versus an undetectable viral load [10 (IQR 4–20) vs. 7.5 (IQR 3.5–18)], *p* = 0.60. Moreover, patients with a CD4 count of less than 200 had a higher score on HIV-SI [12 (IQR 5–27) vs. 10 (IQR 3–18)], *p* = 0.17.

In a multivariate analysis patient age, being female, hepatitis C coinfection, concomitant treatment and non-adherence reported significantly worse tolerance or a higher HIV-SI score (Table 6).

**Table 6:** Multivariate analysis of HIV- SI

		Beta (SD)	*P*-valor
Intercept		3.57 (1.50)	0.018
Age of patient	≤ 43	Reference	-
	43–50	4.39 (1.89)	0.021
	> 50	0.96 (1.93)	0.622
Sex assigned at birth	Male	Reference	-
	Female	4.43 (1.67)	0.009
Coinfection	No	Reference	-
	Yes	4.67 (1.71)	0.007
CD4	< 200	2.09 (2.70)	0.44
	≥ 200	Reference	-
Concomitant treatment	No	Reference	-
	Yes	5.07 (1.56)	0.001
SMAQ Adherence	Yes	Reference	-
	No	5.47 (1.47)	< 0.001

*SD* standard deviation; *SMAQ* Simplified Medication Adherence Questionnaire

### Sensitivity to change of the questionnaire

129 patients answered the HIV-SI and MOS-HIV questionnaire again at a second visit 6 months later. Amongst the group of patients who had not switched treatment, there were no differences in the HIV-SI score from baseline. However, amongst those who had switched treatment, a significantly different score was measured in the pre and post HIV-SI questionnaire. Consequently, HIV-SI does detect differences when the treatment is changed (Table 7).

**Table 7:** Sensitivity to change of HIV- SI questionnaire

	HIV-SI baseline Mean(SD)	HIV-SI difference from baseline Mean(SD)	Cohen Effect Size	SRM	MCID	%MCID
Total (*N* = 129)	13.51(12.30)	0.13(11.68)	0.0106	0.0111	-1.61	43.41
No switch group (*n* = 100)	12.60(12.25)	-0.36(10.24)	-0.0294	-0.0352	1.73	36.00
Switch group (*n* = 29)	16.66(12.17)	1.83(15.78)	0.1495	0.1153	-7.88	37.93

*SD* standard deviation; *SRM* Standardized Response Mean; *MCID* Minimal Clinically Important Difference

## Discussion

In the current study, the Spanish HIV-SI questionnaire showed a high internal consistency (Cronbach’s α of 0.89) and an adequate construct validity (CFI and TLI > 0.90). Contrast with the MOS-HIV questionnaire showed a good association also with mental and physical score (*p* < 0.0001). Moreover, in our study the presence and distress of each symptom was associated with worsened quality of life on the MOS-HIV questionnaire, as occurred in the Justice´s original validation [17]. It is a short, easy to apply index and showed a good comprehensiveness and comprehensibility from the perspective of the patients [24].

The median number of symptoms reported by patients was 5.5 (IQR 3–9), less than in the study by Justice, [17] which had a median of 15 symptoms (IQR 8–19). The most frequently reported symptom in HIV-SI was nervousness and anxiety; followed by fatigue or energy loss, feeling sad or depressed; bloating or stomach pain; and difficulty sleeping. In the original questionnaire, Justice reported similar common symptoms as being fatigue (81%), diarrhea (77%), anxiety (77%), sadness (76%) and difficulty sleeping (76%), but the frequency was higher than in our cohort, probably related to a lesser use of PI or better tolerance with current treatments.

In a recent systematic review by Wang [34] that sought to summarize and categorize the validated HIV-specific PROMs in adults living with HIV and AIDS using COSMIN methodology [35], the HIV-SI received a Class A recommendation, showing good psychometric properties. Nonetheless, some items showed significant differential item functioning amongst different cultural groups indicating insufficient cross-cultural validity [36]. Statistically significant differences were observed for fatigue, fevers, anxiety, and headaches. In our case, despite the existence of a translation of the questionnaire into Latin American Spanish, we decided to validate it in our Spanish HIV population to confirm its functioning and cultural adaptability.

Cahn et al. [37] used the HIV-SI in 80 patients to compare the efficacy and safety of continuing with or switching from a ritonavir-boosted PI (PI/r) and two NRTIs to lopinavir/ritonavir (LPV/r) monotherapy. The baseline score was between 31.7 and 31.8, higher than our study, probably related to PI/r use. In the switching group, 360 days later, the HIV-SI score fell from 31.7 to 26.2 (*p* = 0.003).

Gathe et al. [38] applied HIV-SI to compare the tolerability of switching the HIV treatment from a PI/r-based regimen to the single-tablet integrase inhibitor-based regimen. At Week 4, the switched group had lower prevalence of five symptoms: diarrhea/loose bowels, bloating/pain/gas in stomach, pain/numbness/tingling in hands/feet, nervousness/anxiety, and trouble remembering. In our case, in the subgroup of patients who had switched treatment, a significantly better score was also detected in the HIV-SI questionnaire after switching.

Some studies have made alterations to the original questionnaire. Edelman et al. [39] only considered symptoms reported as “bothering me a little, bothering me or bothering me a lot”, but not symptoms that do not bother. Marc et al. [40] transformed the HIV Symptom Distress Module into a range of 0 to 100, multiplying it by a constant of 25 to create a new summative ratio scale and non-response was interpreted as never experiencing the symptoms or non-occurrence. As in our study, they concluded that male study participants reported a significantly lower symptom distress score than female participants.

A more recent study reported a mean number of symptoms of 9.7 (± 5.4, range 2 to 20) in a sample of 2,000 patients with 76.0% of patients on an INSTI-based regimen [41]. It also identified four clusters of symptoms: a gastrointestinal cluster, a psychological cluster, a pain cluster and a body-image cluster. Another systematic review that included 13 studies with a different symptom assessment tool also defined five of the most reported symptom clusters [42].

There were several limitations to our validation study; for example, the sample is not large enough to detect a difference in adherence assessed by pharmacy record and we have not been able to establish any adherence cut-off related to the symptom score. Another limitation of this study is the lack of comparison between different treatments, although we did confirm the sensitivity to change of the questionnaire in a second visit 6 months later. Future studies on HIV-SI are needed to confirm this point and to assess its applicability and the ability to compile all the patient’s symptoms over time, especially new ones. In addition, cross-cultural validity needs to be improved.

The goal is that application of the HIV-SI test in daily clinical practice may help to improve the detection and management of treatment-related adverse effects, beyond clinical judgment. We therefore believe that the questionnaire should be kept in its original form by adding an open question so that patients can add any other symptoms or experiences they consider important. Therefore, future studies are needed to confirm the sensibility to change, the capacity to detect differences between treatments and its validity when grouping the symptoms in cluster.

## Conclusions

Our study reports the validation of the original HIV Symptom Index in the Spanish PLWHA population. The HIV-SI is a valid, useful and reliable self-administered PROM for routine use to detect and manage HIV related symptoms in clinical practice.

## Electronic supplementary material

Below is the link to the electronic supplementary material.

Supplementary Material 1

## Data Availability

All data generated or analyzed during the study are included in this published article.

## Abbreviations

AEMPS: Spanish Agency for Medicines and Health Products/ Agencia Española de Medicamentos y Productos Sanitarios
AACTG: American Adult AIDS Clinical Trials Unit Outcomes Committee
ART: Antiretroviral therapy
CFA: Confirmatory factor analysis
CFI: Comparative fit index
HCV: Hepatitis C virus
HIV: Human Immunodeficiency Virus
HRQOL: Health-related quality of life
ICC: Intraclass correlation coefficient
LPV/r: Lopinavir/ritonavir
MOS-HIV: HIV Medical Outcomes Survey
MPR: Medication possession ratio
MSAS: Memorial Symptom Assessment Scale
NRTI: Nucleoside reverse transcriptase inhibitors
PI/r: Ritonavir-boosted PI
PLWHA: People living with HIV/AIDS
PRO: Patient-reported outcome
PROM: Patient-reported outcome measure
RMSEA: The root mean squared error of approximation
SMAQ: Simplified Medication Adherence Questionnaire
SDM: Symptoms Distress Module
SSC-HIVrev: Revised Sign and Symptom Check-list for HIV
TLI: Tucker-Lewis Index
VAS: Visual Analogue Scale
VFS: VAS Frequency Scale
VTS: VAS Tolerance Scale
