# Supplementary material for: Psychometric characteristics of the Spanish version of the HIV Symptom Index
Source: J Patient Rep Outcomes. 2024 Oct 1;8:116. doi: 10.1186/s41687-024-00780-2 (PMC11445215; doi:10.1186/s41687-024-00780-2)
Supplement: Supplementary file 1 — Supplementary Material 1 [file 41687_2024_780_MOESM1_ESM.docx]

**Supplementary Table 1. Patients’ demographic and clinical characteristics differences between those who have completed the HIV-SI questionnaire and those who have not.**

|  | **HIV-SI completed**  **n=232(%)** | **HIV-SI not completed**  **n=29(%)** | **Total**  **n=261(%)** | **p-value** |
| --- | --- | --- | --- | --- |
| **Sociodemographic variables** |  |  |  |  |
| Age (years) |  |  |  | 0.1585 |
| - <43 | 77(33.19) | 7(24.14 | 84(32.18) |  |
| - >=43 y <=50 | 77(33.19) | 7(24.14) | 84(32.18) |  |
| - >50 | 78(33.62) | 15(51.72) | 93(35.63) |  |
| Sex assigned at bird |  |  |  | 0.5018 |
| - Male | 174(75) | 20(68.97) | 194(74.33) |  |
| - Female | 58(25) | 9(31.03) | 67(25.67) |  |
| Education level |  |  |  | 0.6840 |
| - No studies | 12(5.17) | 1(3.45) | 13(4.98) |  |
| - Elementary school | 83(35.78) | 12(41.38) | 95(36.40) |  |
| - High school | 59(25.43) | 10(34.48) | 69(26.44) |  |
| - University degree | 50(21.55) | 4(13.79) | 54(20.69) |  |
| - Tercer ciclo | 9(3.88) | 0 | 9(3.45) |  |
| - Unknown | 19(8.19) | 2(6.90) | 21(8.05) |  |
| Work situation |  |  |  | 0.7995 |
| - Unknown | 8(3.45) | 1(3.45) | 9(3.45) |  |
| - Working | 119(51.29) | 13(44.83) | 132(50.57) |  |
| - No working | 105(45.26) | 15(51.72) | 120(45.98) |  |
| Transmission route |  |  |  | 0.0948 |
| - Unknown | 38(16.38) | 9(31.03) | 47(18.01) |  |
| - Homosexual MSM | 67(28.88) | 4(13.79) | 71(27.20) |  |
| - Heterosexual | 66(28.45) | 6(20.69) | 72(27.59) |  |
| - ExUDVP/ Sharing injection material | 56(24.14) | 8(27.59) | 64(24.52) |  |
| - Others (mother to child, transfusion) | 5(2.15) | 2(6.90) | 7(2.69) |  |
| **Clinical variables** |  |  |  |  |
| Duration of infection, years* | 12.37 (8.77)  11.12[4.61-19.39] | 16.40(10.24)  18.64[6.51-22.95] | 12.81(9.00)  11.60[4.75-20.77] | 0.0580 |
| CD4* | 663.52(348.48)  624.5[420-870] | 693.38(270.27)  695[480-840] | 666.84(340.37)  630[426-869] | 0.4119 |
| CD4 cell count, cells/mm^3^ |  |  |  | 0.6552 |
| - <200 | 19(8.19) | 1(3.45) | 20(7.66) |  |
| - 200 – 499 | 64(27.59) | 8(27.59) | 72(27.59) |  |
| - >= 500 | 149(64.22) | 20(68.97) | 169(64.75) |  |
| HIV VL |  |  |  | 1.000 |
| - Undetectable | 208(89.66) | 26(89.66) | 234(89.66) |  |
| - Detectable | 24(10.34) | 3(10.34) | 27(10.34) |  |
| CDC |  |  |  | 0.0620 |
| - Unknown | 61(26.29) | 12(41.38) | 73(27.97) |  |
| - A | 109(46.98) | 6(20.69) | 115(44.06) |  |
| - B | 21(9.05) | 4(13.79) | 25(9.58) |  |
| - C | 41(17.67) | 7(24.14) | 48(18.39) |  |
| HBV Coinfection |  |  |  | 0.0972 |
| - Unknown | 6(2.59) | 3(10.34) | 9(3.45) |  |
| - No | 166(71.55) | 19(65.52) | 185(70.88) |  |
| - Yes | 60(25.86) | 7(24.14) | 67(25.67) |  |
| **Treatment variables** |  |  |  |  |
| Treatment experience |  |  |  | 0.0103 |
| - Unknown | 5(2.16) | 2(6.90) | 7(2.68) |  |
| - Naive | 62(26.72) | 1(3.45) | 63(24.14) |  |
| - Pretreated | 165(71.12) | 26(89.66) | 191(73.18) |  |
| Years taking antiretroviral therapy |  |  |  | 0.1457 |
| - Unknown | 19(8.19) | 6(20.69) | 25(9.58) |  |
| - <=5 | 85(36.64) | 6(20.69) | 91(34.87) |  |
| - 5-10 | 47 (20.26) | 5(17.24) | 52(19.92) |  |
| - 10-20 | 62 (26.72) | 10(34.48) | 72(27.59) |  |
| - >20 | 19 (8.19) | 2(6.90) | 21(8.05) |  |
| Years taking current treatment* | 3.17 (2.78)  2.57 [0.83-4.91] | 2.56(2.35)  2.04[0.59-3.68] | 3.11(2.74)  2.49[0.81-4.90] | 0.3373 |
| Number of ART daily pills * | 2.77(1.66)  3[1-3] | 3.38(1.45)  3[3-4] | 2.84(1.65)  3[1-3] | 0.0197 |
| ART Daily intake |  |  |  | 0.1499 |
| - Bid | 52(22.41) | 10(34.48) | 62(23.75) |  |
| - Qd | 180(77.59) | 19(65.52) | 199(76.25) |  |
| HCV treatment | 7(3.02) | 0 | 7(2.68) | 0.5585 |
| Others concomitant treatment | 120(51.72) | 18(62.07) | 138(52.87) | 0.3411 |
| **Adherence** (%MPR)* | 95.16(8.15)  100[94-100] | 96.24(6.04)  99[95-100] | 95.28(7.94)  100[94-100] | 0.9405 |
| Adherence interval in days* | 245.82(188.61)  180[180-294] | 212.48(72.59)  196[180-236] | 242.06(179.53)  180[180-274] | 0.9019 |
| Adherence MPR ≥90% | 197 (84.91) | 25(86.21) | 222(85.06) | 1.000 |
| Adherent with SMAQ | 131(56.47) | 17(58.62) | 148(56.70) | 0.8457 |

ART: antiretroviral therapy; MSM, men who have sex with men; IDU: intravenous drug user; VL: viral load; bid: twice a day; qd: once a day; IQR: interquartile range; SD: standard deviation; MPR: medication possession ratio; SMAQ: Simplified Medication Adherence Questionnaire; NRTI: nucleoside reverse transcriptase inhibitors; NNRTI: non-nucleoside reverse transcriptase inhibitor; bPI: boosted protease inhibitor; INSTI: integrase strand transfer inhibitor. *mean (±SD); Median [IQR25-75]
